# Source Data Verification (SDV) quality in clinical research: A scoping review

**DOI:** 10.1017/cts.2024.551

**Published:** 2024-05-21

**Authors:** Muayad Hamidi, Eric L. Eisenstein, Maryam Y. Garza, Kayla Joan Torres Morales, Erika M. Edwards, Mitra Rocca, Amy Cramer, Gurparkash Singh, Kimberly A. Stephenson-Miles, Mahanaz Syed, Zhan Wang, Holly Lanham, Rhonda Facile, Justine M. Pierson, Cal Collins, Henry Wei, Meredith Zozus

**Affiliations:** 1 University of Texas Health Science Center at San Antonio, San Antonio, TX, USA; 2 Duke University, Durham, NC, USA; 3 University of Arkansas for Medical Sciences, Little Rock, AR, USA; 4 University of Vermont, Vermont Oxford Network, Burlington, VT, USA; 5 Janssen R&D LLC, Raritan, NJ, USA; 6 ICON PLC employee on an assignment to Janssen R&D LLC, Dublin, Ireland; 7 OpenClinica, Needham Heights, MA, USA; 8 Regeneron, Tarrytown, NY, USA; 9 Clinical Data Interchange Standards Consortium (CDISC), Austin, TX, USA

**Keywords:** Clinical research, clinical trial monitoring, quality, Source Data Verification

## Abstract

**Introduction:**

The value of Source Data Verification (SDV) has been a common theme in the applied Clinical Translational Science literature. Yet, few published assessments of SDV quality exist even though they are needed to design risk-based and reduced monitoring schemes. This review was conducted to identify reports of SDV quality, with a specific focus on accuracy.

**Methods:**

A scoping review was conducted of the SDV and clinical trial monitoring literature to identify articles addressing SDV quality. Articles were systematically screened and summarized in terms of research design, SDV context, and reported measures.

**Results:**

The review found significant heterogeneity in underlying SDV methods, domains of SDV quality measured, the outcomes assessed, and the levels at which they were reported. This variability precluded comparison or pooling of results across the articles. No absolute measures of SDV accuracy were identified.

**Conclusions:**

A definitive and comprehensive characterization of SDV process accuracy was not found. Reducing the SDV without understanding the risk of critical findings going undetected, i.e., SDV sensitivity, is counter to recommendations in Good Clinical Practice and the principles of Quality by Design. Reference estimates (or methods to obtain estimates) of SDV accuracy are needed to confidently design risk-based, reduced SDV processes for clinical studies.

## Introduction

Clinical trial complexity continues to rise and increases the effort required at clinical investigational sites [1–5]. This has contributed to clinical trial operational inefficiency being considered one of the major impediments to Clinical and Translational Research [6]. The rate of clinical trial cost increase – driven by study complexity and operational inefficiency – is greater than inflation for other segments of the economy [7–9]. An estimated 46% percent of on-site monitoring time has been attributed to Source Data Verification (SDV) [10]. Estimates for the portion of clinical trial costs attributable to SDV range from 25% to 40%, implicating SDV as a major cost driver [10–13]. Reducing the amount of manual SDV in clinical trials would create an opportunity to increase operational efficiency and lower clinical trial costs.

The comparison of study data to the medical record (or other sources) to verify that the medical record data are accurately reflected in the study data is called SDV (definitions Table 1). Since the earliest reported use in the year 1746 [14], SDV has been used as a tool to find and fix errors from medical record abstraction (MRA), the process associated with the largest error rate in data processing [15].

**Table 1.** Key definitions relevant to source document verification

**Monitoring**: The act of overseeing the progress of a clinical study, and ensuring that it is conducted, recorded, and reported in accordance with the protocol, standard operating procedures, Good Clinical Practice (GCP), and the applicable regulatory requirements [16]. Clinical study monitoring includes activities such as ensuring good communication between site investigators and the Sponsor, verifying adequate resources, storage, and accountability of the investigational product and biological samples, Informed Consent, and regulatory compliance and protocol adherence at sites, ensuring that site personnel is qualified for their roles on the study, tracking recruitment, enrollment, and retention, ensuring appropriate reporting of adverse events, deviations and problems, and ensuring the completeness and accuracy of study data through SDV [17]. **Risk-based monitoring**: an adaptive approach to clinical trial monitoring that directs monitoring focus and activities to the evolving areas of greatest need that have the most potential to impact subject safety and data quality [18]. **On-site monitoring**: An in-person evaluation carried out by sponsor personnel or representatives at the sites at which the clinical investigation is being conducted [19]. In risk-based approaches, on-site monitoring may be focused on activities that are critical to safety or study results; this is referred to as *Targeted On-site Monitoring* [16]. **Off-site monitoring**: Remote monitoring via telephone and email without visiting the site institutions [20]. **Centralized monitoring**: (1) A remote evaluation carried out by sponsor personnel or representatives at a location other than the sites at which the clinical investigation is being conducted [19]. (2) Document review, data review, and analysis performed remotely from the investigator site to examine the data collected to check compliance and identify unusual patterns and deviations [21]. This is also called *remote monitoring*. **Source data verification (SDV)**: the comparison of study data to their original recording to ensure that, “the reported trial data are accurate, complete, and verifiable from source documents [16].” Source Document Review [18] describes the review of source documents for protocol adherence, quality of documentation, as well as site processes in contrast to transcription checking, referred to as Source Data Verification (SDV). **Targeted SDV**: A risk-based approach that focuses SDV efforts toward data that are critical to safety or study results. Targeted SDV may result in fewer data fields being verified and is sometimes called *Reduced SDV*. **Reduced SDV**: a decrease in the amount of SDV performed for a study through performing SDV for some (or none) of the sites, patients, visits, data elements, or data values. **Quality by design**: A systematic approach, “that begins with predefined objectives and emphasizes product and process understanding and process control, based on sound science and quality risk management [22].”

**Figure 1.**
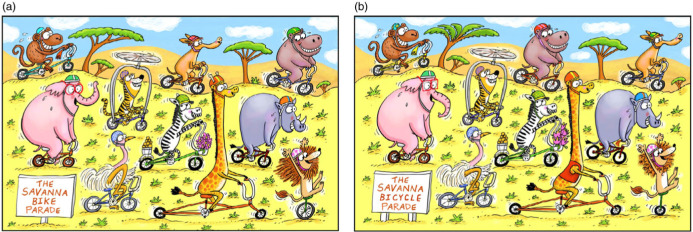
Visual inspection exercise. © 2020 Highlights for Children, Inc. All rights reserved. Permission to reproduce and distribute this page is granted by Highlights for Children.

Extensive and often 100%, SDV was historically considered necessary to ensure study data quality and has served as the foundation upon which trialists claimed data accuracy and authenticity [23,24]. Cognitively similar to MRA, SDV is a manual inspection process performed by humans, and the error rate is likely similarly high. Finding the twenty-one differences between Figure 1a, b illustrates challenges with SDV as a mechanism to identify data errors.

Decades ago, the field of HRA demonstrated that errors in the manual inspection process had a significant and often overlooked impact on the average outgoing quality [25]. SDV has long been used as a tool to assure data quality. However, the quality of this tool itself was rarely examined. Ironically, maintaining a current calibration record for all devices utilized in a clinical trial is required, while no comparable requirement exists for the more fallible processes of manual abstraction from medical record sources and manual inspection by SDV. Though at reduced levels through risk-based approaches, SDV is still being used as a major strategy to identify errors and ensure high-quality data [26–28]. This thinking must change. As a fallible manual process, SDV was never capable of assuring high-quality data from a fallible source [25,29]. SDV provided a convenient answer, “all data were verified against the source,” but the assumption that often followed, “therefore the data are correct,” was flawed. This leaves us with the uncomfortable difficulty of needing to verify the accuracy of data in the absence of a true gold standard, a conundrum that likely perpetuated reliance on manual SDV.

Reports of SDV error and omission started to emerge with studies evaluating various risk-based monitoring approaches[30,31], and along with them, acknowledgment that manual SDV processes are not capable of producing error-free data. In this context, the value of the traditional 100% SDV has increasingly been questioned [11,12,32–34]. In a recent national study, respondents characterized the “amount of money, time and resources spent on certain monitoring methods” such as extensive on-site SDV, as being wasteful for commercial trials [35]. Federally funded and investigator-initiated studies traditionally employed more limited monitoring approaches due to budget constraints [36–39]. With the shift toward risk-based approaches articulated in the United States Food and Drug Administration’s (FDA’s) guidance on risk-based monitoring in 2013 [19] and the 2018 revision of the Good Clinical Practice (GCP) guidelines [40], industry and academic monitoring practices are converging with risk-based approaches now strongly encouraged by prominent industry groups [18,41,42] and regulators [19,21,43]. Prior to the COVID-19 pandemic, however, industry adoption of risk-based monitoring practices, such as off-site remote monitoring, reduced Source Document Review (SDR), reduced SDV, and centralized monitoring (definitions in Table 1), remained low, 25%, 16%, 20%, and 16% respectively [44].

Only moderate evidence supports the comparability of traditional (100%) and reduced SDV [27]. European Medicines Agency (EMA) has reached beyond the status quo and called for demonstration that alternate monitoring methods are non-inferior to traditional methods [45]. GCP guidelines and the FDA, through Quality by Design (QbD) principles [16,41], have communicated general heightened expectations for quality planning and the design or selection of study processes with the capability to deliver the data accuracy required to support planned study analyses. However, computational models for the *a priori* design of capable reduced SDV processes are not common, and the inputs needed for the computational models, such as error rates for SDV processes, have not been widely reported. The resulting uncertainty in process capability is a likely contributor to the slow adoption of reduced SDV, along with a lack of comparative evidence, lingering concerns of feasibility, the effort required to change existing organizational processes, and methodological questions regarding reduced SDV. Filling these knowledge gaps will provide the means to design and optimize SDV for clinical studies. We conducted this review to minimize this knowledge gap.

## Objective

The goals of this scoping review were (1) to identify published reports of SDV quality and (2) to characterize and summarize the evidence regarding the quality of the SDV performed in clinical studies.

## Methods

The Preferred Reporting Items for Systematic Reviews and Meta-analysis extension for Scoping Reviews (PRISMA-ScR) [46] methodology was adopted (Supplementary Material 1 - PRISMA-ScR-Fillable-Checklist). The protocol components, as outlined in the PRISMA-Scr, were standardized *a priori* and used by authors, though the protocol was not registered in a review protocol registry such as Open Science Framework.

### Eligibility criteria

Studies were included if (1) the main focus was the quality of the SDV process, i.e., problems with or ways to achieve the quality of the SDV process, (2) the full-text article was available, and (3) the article was written in the English language. Studies were excluded if they (1) were not focused on SDV quality, (2) were not full-text peer-reviewed articles, e.g., abstracts, posters, regulations, and policies, or (3) were not in English. All types of study designs were included; however, policy documents, guidelines, and regulations were excluded.

### Information sources

The following databases were searched: MEDLINE, CINAHL, PsycINFO, Embase, Web of Science, Scopus, Cochrane, The Association of Clinical Research Professionals, The Society of Clinical Research Associates, Applied Clinical Trials (ACT), and Google Scholar were used as a safety net to confirm that our search process didn’t miss any relevant papers. The last search was executed in October 2022. In addition, the list of similar articles is suggested by the NLM website next to each search result.

### Search

The following PubMed query was used: (“SDV”[Title/Abstract] OR “clinical trial monitoring”[Title/Abstract]) AND (“clin res”[Journal] OR “clinical research”[Title/Abstract] OR “clinical trial”[Title/Abstract] OR “clinical study”[Title/Abstract]) with the syntax modified as needed to run on the other databases.

*Selection of sources of evidence* the database searches were performed by three authors (MM, KTM, MNZ), with the initial screening of titles and abstracts performed by two authors (MM, MNZ). The articles that passed the initial screening were retrieved for full-text review. Two independent people reviewed the full text of the retrieved articles and attempted consensus on disagreements. Disagreements for which consensus could not be achieved were adjudicated by a majority vote of authors on weekly adjudication calls.

### Data extraction process

A spreadsheet (Supplementary Material 2 – Information abstracted from articles) was created to capture the data needed to be extracted. All authors independently participated in abstracting a predefined list of relevant Information from each included article.

### Data items

Information abstracted from the included articles is listed in (Table 2), which can be grouped into article metadata, information about research design and context, and information about SDV accuracy.

**Table 2.** Information abstracted from articles

Article metadata/bibliographic information	Year published
	Author’s last name
	Article title
	Volume issue journal name
Inclusion/exclusion	Included or excluded after full-text review
	If excluded after full-text review, list exclusion reasons.
	Group adjudication/final determination
Review assignments	Reviewer 1 name
	Reviewer 2 name
Information about the article or paper	Article reports primary research results (1 = affirmative, 0 = negative)
	Article reports secondary research results, i.e., cites/mentions results reported elsewhere (1 = affirmative, 0 = negative)
	Article reports ONLY opinion or consensus statements as the main focus (1 = affirmative, 0 = negative)
	Purpose of the paper (free text)
	Years over which study was conducted
Information about research design	Research design (experimental, quasi-experimental, pre-exp., descriptive-quantitative, descriptive-qualitative, non-research)
	If experimental; control and comparator?
	If experimental, Allocation method?
	Outcome / dependent variables (i.e., the thing that you expect to change after the exposure or intervention) and operational definitions.
	When with respect to (wrt.) Intervention or exposure were observations made (before, after or both)
	Mostly for experimental, quasi-experimental or pre-experimental studies, were the study Results / interpretation uncertain or clear-cut?
	Study strengths.
	Study weaknesses.
Information about how SDV accuracy was measured in the study	SDV* accuracy measured (1 = affirmative)
	How was SDV accuracy measured
	Unit of analysis for the SDV error rate (data field, record, form/page, visit, research subject, abstractor or monitor, research site, study)
	Number of data values assessed (error rate denominator)
	Number of data errors identified (error rate numerator)
	Accuracy statistics reported (agreement, chance-adjusted agreement, error rate, sensitivity, or specificity)
	Other aspects of SDV quality measured (free text)
	SDV accuracy results interpretation (equivocal vs. clear)
Information about the research context, e.g., type/s of studies in which the research was conducted	Parent study NCT number (if any)
	Parent study type (RCT, registry, correlational, PCT, CER, HSR, epidemiology, other)
	Parent study therapeutic area
	Parent study context – FDA vs. NIH regulated
	Research context - Sponsor-led vs. Investigator-led
	Type of source documents, e.g., paper charts vs. electronic source
	Medical record abstraction quality assurance used (standardized abstraction methods, abstraction training, abstraction environment, abstraction process control via re-abstraction)
	Description of pre-SDV data processing, such as query rules ran prior to SDV (free text)
	Percent SDV
	SDV method (read full chart to confirm representation on study forms, confirmed data on study forms by locating value in the chart, other)
	Who carried out SDV (study coordinator, monitor, auditor, other)
Comments	Free text field for the reviewers to make any comments not represented in other columns
Article acquisition	1 = Obtained; 0 = Unobtainable

CER = Clinical Evaluation Report; FDA = The Food and Drug Administration; HSR = Health Services Research; NCT Number = National Clinical Trial number; NIH = National Institutes of Health; PCT = Pragmatic Clinical Trial; RCT = Randomized Controlled Trial; SDV = Source Data Verification.

### Critical appraisal of individual sources of evidence

As a scoping review, we sought to characterize all available evidence regarding SDV quality. Since we anticipated a wide variety of evidence, we critically apprized the strength of evidence according to the research design and context of the study.

### Synthesis of results

We grouped the studies by study design and context. Examples include prospective *versus* retrospective approach and experimental *versus* quasi-experimental, pre-experimental, or descriptive designs. Use of comparators and control, and whether the study measured SDV discrepancies or SDV errors were also used to categorize studies as well as aspects of the research context such as the number of studies, sites, or participants included in the quality assessment, the therapeutic area in which the SDV quality assessment was conducted, and whether the study was industry-funded.

## Results

### Selection of sources of evidence

**Figure 2.**
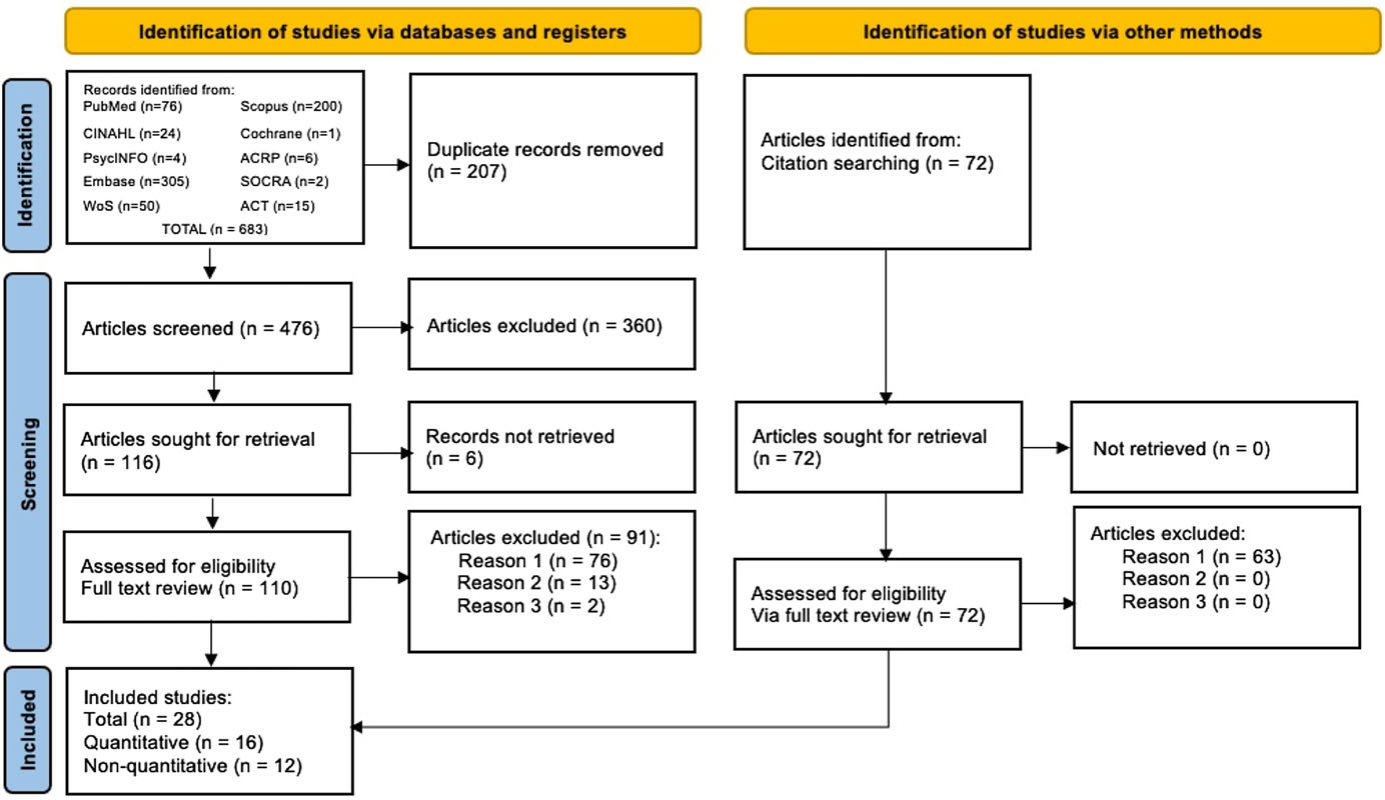
Flow diagram [64]. ACRP = The Association of Clinical Research Professionals; ACT = Applied Clinical Trials; EMBASE = Excerpta Medica database; PsycINFO = American Psychological Association PsycInfo database; SOCRA = The Society of Clinical Research Associates; WoS = Web of Science database.

The search of bibliographic databases yielded 683 records, and an additional 72 were identified from searching citations (paper references). (Fig. 2). From the total of 755 records identified, there were 207 duplicate records removed, 360 excluded per the Title and Abstract screening process, and 6 unretrievable. In total, 182 articles (110 plus 72 in Fig. 2) were retrieved and underwent full-text review. The full-text review eligibility assessment resulted in the exclusion of 154 articles (91 plus 63 in Fig. 2) and the inclusion of 28 articles. Sixteen of the included articles reported quantitative results.[17,18,31,33,37,38,47–56] Twelve articles reported non-quantitative studies [23,26–28,32,57–63] (Fig. 2).

### Characteristics of sources of evidence

#### Quantitative studies

Seven of the included quantitative studies prospectively collected data after assigning the patient, site, or study to some monitoring strategy [17,37,47,48,52,54,55]. Nine analyzed existing data [18,31,33,38,49–51,53,56] (references detailed in Table 3). Ten studies were classified as using pre-experimental designs, two were classified as quasi-experimental, and four were classified as experimental studies (references reported in Table 3; study details reported in Supplementary Material 3).

**Table 3.** Research design summary for the included quantitative studies

Design	Prospective	Retrospective	Total
Experimental	4 [17,37,52,54]	0	4
Quasi-experimental	1 [55]	1 [31]	2
Pre-experimental	2 [47,48]	8 [18,33,38,49–51,53,56]	10
**Total**	7	9	16

#### Non-quantitative evidence

A total of 12 non-quantitative articles were included, nine opinions [23,32,57–63], and three reviews [26–28]

*Opinion*: Two of the nine opinion papers reported a clinical trial quality event that served as a major inflection point in thinking and approach [57,58]. These articles reported National Institutes of Health (NIH) tightening the clinical trials monitoring requirements in response to the finding of fraud in the National Surgical Adjuvant Breast and Bowel Project study group [57,58]. At the time, clinical trial monitoring practices, including SDV, varied considerably across multicenter studies funded by the NIH [58]. Six opinion articles [23,32,34,59–61] provided guidance on different aspects of SDV or practice recommendations to improve the SDV process, multiple of them with the purpose of prompting change. One provided insight into the cost of SDV [63].

*Reviews*: three literature reviews were included [26–28] one review assessed SDV empirically [27] (2021 Klatte) adopted the Cochrane methodology which included only prospective empirical studies. The two remaining included reviews [26,28] focused on SDV practices. Houston et al. (2018) reported a wide range of SDV methods existing in practice with no best practice evident [26]. Similarly, Ward’s review documented the absence of methodological guidelines for SDV, the wide variety in reported practice, and the lack of empirical evidence regarding the impact of SDV [28], which stood out as the earliest identified graduate research presented as a thesis in a master’s degree program.

## Discussion

### Critical appraisal within sources of evidence

In comparison to the Cochrane review, which included only prospective empirical studies, this review includes a much broader range of evidence from retrospective empirical studies, other reviews, and opinion papers. Though we did not explicitly assign a strength of evidence or risk of bias rating, we fully acknowledge that the retrospective, descriptive, and qualitative papers provide inherently weaker evidence due to threats to internal validity and risk of bias. For example, with one exception, there were no measures of SDV quality reported prior to broadened interest in RBM. However, the two-dimensional framework for evaluating SDV quality (Table 3) would not have emerged without considering the outcomes measured in the retrospective studies. As a scoping review, these sources were included to provide a comprehensive compilation of the available literature and findings across the spectrum of research designs while at the same time recognizing the difference in evidence strength.

The recent systematic literature reviews for Good Clinical Data Management Practices also found scant Cochrane-strength evidence for practice recommendations [65–69]. In these reviews, randomized, controlled experiments of operational processes were few and far between. It may be that this standard is not a feasible expectation for evidence-supporting practice in clinical research operations. On the other hand, it still seems ironic that the methods from which strong evidence is generated are not themselves held to that same standard. Without such a standard, some may interpret the literature regarding SDV accuracy as sufficient to recommend against SDV, while others looking at the same literature will refrain from such recommendations.

### Results of individual sources of evidence and synthesis of results

The included quantitative studies exhibited significant heterogeneity along multiple dimensions. We identified four reported aspects of SDV quality (rows in Table 4): (1) source data availability and access, (2) data quality, (3) study process fidelity, and (4) SDV process fidelity. Furthermore, reports for the SDV quality domains naturally fell into three main categories (columns in Table 4): (1) SDV quality measures, such as rates of findings, missing data, or process problems, (2) the impact of SDV on decreasing future problems, and (3) the impact of SDV on study results. One of the included articles assessed SDV quality on all three levels [53]. Three studies reported a measure of source data availability and access [17,51,56] (Table 4). The data quality aspect (domain) of SDV quality (second row in Table 4) was reported as data discrepancies and errors. Six articles reported counts or rates of data discrepancies [17,18,31,47,48,52], while seven reported measures of errors [33,47,49,51,53,55,56]. An error is a discrepancy with the truth, i.e., an incorrect data value. A discrepancy, on the other hand, is a difference between two data values where either or both could be in error. Discrepancies are often reported when the correct value cannot be determined or was not determined. In the included articles, some reported database changes were made after investigating and resolving discrepancies; we counted these as reports of errors.

**Table 4.** Aspects of source data verificaiton (SDV) quality and the level at which they were reported

SDV quality aspects (domains of SDV quality)	Reports of measures in the SDV quality domain	Reports of SDV preventing future occurrences	Reports of SDV impact on study results
**Source data availability and access**	3 [17,51,56]	–	–
**(2) Data quality (DQ)** *			
Discrepancies	6 [17,18,31,47,48,52]	–	3 [31,48,52]
Errors	6 [33,47,49,53,55,56]	1 [53]	2 [33,51,53]
**(3) Study process fidelity** * ^,^ †			
Human subject protection	–	–	–
IRB oversight	1 [38]	–	–
Informed consent	6 [37,38,52,54”^56^]	1 [37]	1 [56]
Privacy	–		1 [54]
Safety	8 [37,38,47–49,51,54,55]	1 [37]	4 [47,48,51,54]
Site study team training	–	–	–
Research subject disposition	–	–	–
Identification and screening	–	–	–
Eligibility	6 [37,38,48,52,54,56]	1 [37]	3 [48,54,56]
Enrollment	–	–	–
Allocation/Exposure	1 [38]	–	–
Retention	1 [54]	–	–
Completion/Withdraw	1 [54]	–	1 [48]
Protocol Adherence**	4 [37,48,55,56]	1 [37]	5 [47,48,52,54,56]
Investigational product	1 [38]	–	- -
Essential documents	1 [38]	–	–
Unanticipated problem identification and handling		–	–
Unspecified audit findings	1 [50]	–	–
Unsubstantiated data alteration or fraud	–	–	–
**(4) SDV process fidelity**	1 [49]	–	–
**Number of Distinct Articles Reporting at Each Level**	**16**	**2**	**9**

* These assessments were done relative to another method rather than as a quantification of absolute errors. i.e., the studies have not measured SDV actual accuracy, instead, surrogates for accuracy were measured, such as a number of data discrepancies or errors missed relative to some other method.

† Includes delivery of the intervention, endpoint Assessment, regulations, guidance, and other requirements to which the protocol must comply.

**Figure 3.**
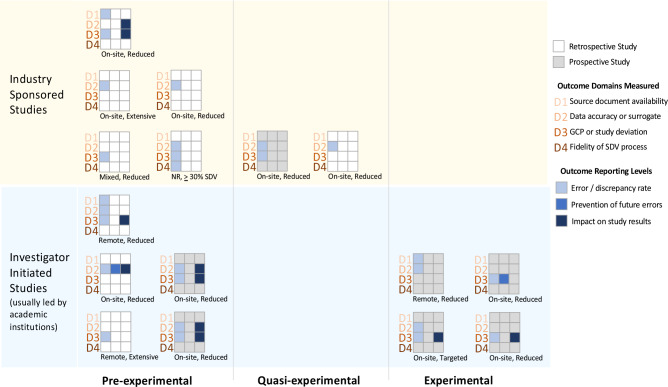
Heterogeneity in SDV* quality assessment. Each 3x4 grid in the figure represents SDV quality assessments reported in one included, quantitative article. The SDV methods compared are listed at the bottom of each grid, with NR signifying not reported, extensive signifying high amounts of data values undergone up to 100% SDV, and mixed signifying a combination of two or more SDV methods. *Source Data Verification (SDV): the comparison of study data to their original recording to ensure that, “the reported trial data are accurate, complete, and verifiable from source documents [16].”

The third and most frequently reported aspect of SDV quality was the ability to detect or propensity to miss study process errors; we refer to this as study process fidelity (Table 4). The included articles varied greatly concerning the study processes for which SDV quality was reported. Eight articles reported SDV quality with respect to the detection of safety issues [37,38,47–49,51,54,55]. One reported that SDV improved safety process fidelity over time [37]. And four reported the impact of SDV on safety-related study results [47,48,51,54]. Eligibility, informed consent, and protocol adherence were tied as the second most frequently reported aspects of study process fidelity (Table 4). These aspects encompass the EMA (2017) integrated inspection report categories used to summarize and evaluate the potential implications of major or critical findings, “the impact on the integrity of the trial data, the rights, wellbeing, and safety of the subjects, the compliance of the trial with GCP (including ethical principles) …” [70]. Within each SDV quality aspect, the included articles varied in whether and, if so, how the impact of SDV was evaluated. All sixteen included quantitative articles reported counts or rates of items detected or missed by SDV (Table 4, Fig. 3). Eleven [17,18,31,33,47–49,52,53,55,56] of the sixteen quantitative articles reported measures of data discrepancies or errors. Two articles [37,53] evaluated the impact of SDV on quality improvement over time within a study, whereas nine [31,33,47,48,51–54,56] evaluated the impact of SDV findings on study results (Table 4, Fig. 3). However, five [31,33,52,54,56] of the articles reporting the impact of SDV findings on study results did so qualitatively, for example, stating that the errors occurred evenly across treatment groups, that the errors occurred in non-critical variables, or that the frequency or extent of the errors was too small to have impacted the analysis. The remaining four [47,48,51,53] articles reporting the impact of SDV findings on study results did so by comparing analyses before and after error correction.

In addition to variability in the aspects of the SDV quality measured and the level at which they were reported (Table 4), the included articles exhibited just as much variability in the context in which they were measured. For example, seven assessments were done in industry clinical trials versus nine in investigator-initiated studies (Fig. 3). The included articles were heterogeneous with respect to the SDV process for which the quality was measured. The most common SDV process variants assessed included remote, targeted, and varied extents of reduced SDV (Fig. 3, Supplementary Material 3). All reported SDV quality assessments were relative, for example, comparing the number of SDV findings missed by one method that was subsequently detected by another, or reporting the portion of SDV findings that resulted in database changes. The reported assessments differed in how discrepancies or errors were identified, i.e., which two SDV processes were compared to identify items missed by one of the methods but detected by the other. The assessments differed in whether these discrepancies were verified. Multiple included studies reported the number of data discrepancies detected by SDV, while others reported those missed by SDV. One study [37] comprehensively reported the number of discrepancies detected and missed by SDV but did so for only one parameter, informed consent (Supplementary Material 3). Further limiting the quantitative synthesis, the unit of analysis was inconsistent across included studies and included counts of individual findings, rates of findings per patient, rates of findings per site, or proportions of patients or sites with one or more findings. Additionally, some studies reported only major findings, while others reported all findings. These differences are detailed in Supplementary Material 3. Although multiple studies quantified one or more aspects of SDV accuracy, no article reported a comprehensive measure of SDV accuracy. No study reported the absolute rate of errors, i.e., detected against the gold standard of truth. One study [52] declared 100% SDV to be the gold standard but did not compute accuracy measures (sensitivity and specificity) against it.

The search strategy broadly encompassed articles focused on SDV as well as those focused on clinical study monitoring; relevant reports of SDV quality were found in both types of articles. For example, a study comparing 100% SDV *versus* reduced SDV that quantified items missed by 100% SDV provided an assessment of SDV quality. Similarly, a study comparing triggered *versus* non-triggered on-site visits that quantified items missed by SDV provided an assessment of SDV quality.

The majority of included articles reported SDV quality in the context of comparing RBM (including targeted, remote, or reduced SDV) to traditional monitoring approaches usually characterized by more extensive SDV. The heterogeneity, such as differences in the amount, timing, or frequency of SDV, in articles included in the quantitative synthesis (Table 4 and Supplementary Material 3) means that the assessments of SDV quality are not comparable; the methodological heterogeneity limited this synthesis to a scoping review.

The evaluative studies identified and included in this review are varied in terms of which domains of SDV quality are reported for SDV (rows in Table 4). Reported SDV quality domains included accessibility of source data, data error rates, GCP or protocol deviations (often called monitoring findings), and audit-identified deviations in the SDV process. Further, these SDV quality domains were reported at multiple levels, including accessibility of source data needed to identify errors, rates of identified errors, effectiveness at preventing future errors, and impact of the identified errors on study outcomes (columns in Table 4). SDV likely has utility on each level, however, reports at the prevention and study results levels were few compared to counts or rates of findings. A comprehensive evaluation of SDV quality would include the rows and columns in Table 4 as well as items both identified and missed by SDV.

SDV accuracy cannot be calculated without a gold standard and enumeration of true positives, false positives, true negatives, and false negatives. One study [52] declared 100% on-site SDV as the gold standard; however, the authors acknowledge that errors remain in 100% SDV’d data and did not report accuracy measures of sensitivity and specificity. Thus, we did not find a quantitative report of absolute SDV accuracy (sensitivity and specificity).

### Related findings

**Figure 4.**
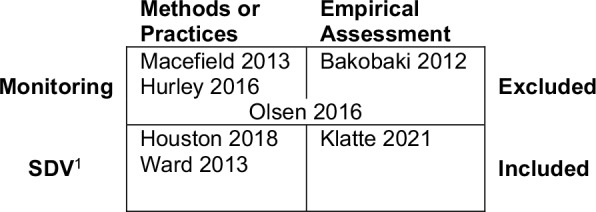
Categorization of review articles. ^1^Source Data Verification: the comparison of study data to their original recording to ensure that, “the reported trial data are accurate, complete, and verifiable from source documents [16].”

There is a good number of reviews and surveys. Despite the fact that they didn’t meet the inclusion criteria, they reported related beneficial results, and we opted to present them. Seven relevant reviews [11,26,27,71–73] were identified by the search (Fig. 4). Three of the identified reviews met the inclusion criteria (bottom row in Fig. 4). The reviews differed in their scope, with the three included reviews specifically addressing SDV and the four excluded reviews focusing more broadly on clinical trial monitoring or other aspects thereof (Fig. 4). Four of the reviews focused on methods; two included only empirical assessment of monitoring or SDV outcomes, and one review, Olsen et al. (2016), included both methods and empirical results (Fig. 4). While the Ward (2013) survey did not meet inclusion criteria, the systematic review portion of this work did and is included (Fig. 4). Only the three reviews, including Ward (2013), specifically addressed SDV were included in this review.

The Cochrane review by Klatte et al. (2021) included eight prospective empirical studies that compared different monitoring strategies [27]. Five of them are also included in our review. Overall, the Cochrane review concluded with moderate certainty that “risk-based monitoring is not inferior to extensive on-site monitoring with respect to critical and major monitoring findings in clinical trials” and noted that “more high-quality monitoring studies that measure effects on all outcomes specified in this review are necessary to draw more reliable conclusions.” The Cochrane review did not directly address the accuracy of the SDV process itself with respect to the identification of errant data.

Eight surveys [13,28,74–79] were identified through the search, all but one [28] were excluded due to minimal focus on SDV quality. However, because each survey contained one or more SDV-relevant questions, we have listed them in Supplementary Material 4 for completeness. Across the surveys, perceptions varied widely with respect to the impact of SDV or monitoring on data quality. Collectively, the survey work indicates variability in SDV frequency, amount, and methods similar to that seen in the quantitative, included articles and in the included reviews. Multiple articles reporting survey results called for additional research on SDV methods, the impact of SDV, and more specific guidelines for methods, including the amount of SDV. However, their message is weakened by the limited generalizability of each survey and the significant changes in context and practice over the almost 30-year span over which survey results were reported.

Evidence throughout the literature, from opinion to experimental studies, supports the ability of SDV to identify unreported events. While reports of SDV-identified significant or systematic findings certainly exist [47,48,51,52], the included articles reporting the impact of missed-event-type findings on study results indicated no significant impact [27,37,51]. Similarly, multiple included studies concluded a lack of impact of SDV-identified data errors on study results[31,48,51]. However, others found SDV-identified data discrepancies or errors impactful on one or more study analyses [17,53]. These apparent differences may reflect differences in the SDV quality domains assessed, differences in measurement methods, differences in the SDV processes themselves, or differences in other aspects of data collection and processing. For example, one of the included studies assessed the type, frequency, and impact of data errors on an observational study and concluded that data errors identified through SDV would have otherwise impacted the study results [53]. However, few upstream data quality control measures were in place. Similarly, two articles concluding no impact of SDV-identified data error measured SDV error for query-clean data and data collected on structured site worksheets (rather than abstracted from medical records) – the upstream data quality control measures described would have likely significantly decreased the number of errors remaining to be detected by SDV. The many-faceted heterogeneity precludes drawing conclusions.

With rising cost pressure, a wide variety of options continue to be explored. The Cochrane review concludes, albeit on what the authors deem to be moderate to low-quality evidence, that there is likely no difference between the different monitoring (and SDV) strategies tested in the five comparisons assessed by the review. We posit that this could be due to the likely high error rate of SDV itself, i.e., less of an error-prone inspection may not yield markedly worse overall quality. One of the most convincing studies comparing the outgoing error rate from RBM to traditional monitoring concluded the same, noninferiority [80].

Remote access to Electronic Health Records (EHRs) for monitoring and the ability to extract data directly from them greatly impact study monitoring and SDV processes. The justification for pursuing such EHR-to-eCRF data collection includes increasing data quality by decreasing manual medical record abstraction and decreasing the data collection burden at sites and Sponsors. In this case, SDV can be performed by computationally confirming that study data match the EHR source or may obviated altogether as mentioned in the FDA eSource guidance [81]. Eliminating manual SDV for EHR-to-eCRF data would likely reduce burden and cost. Given advances in technology and permissible regulatory guidance, available innovation will likely be applied to SDV, such as (1) establishing traceability back to the source that can be computationally traversed to demonstrate that the final data exactly reflects the medical record source and (2) defining a certified copy in the context of data extracted from EHRs such that sponsors and regulators are guaranteed that the final data are a replica of the source. These cannot be accomplished with manual SDV.

### Limitations

Though performed systematically, our search could have missed an important article. The database searches were conducted over a 5-month period which may result in differences (though minimal) of returned articles. The methodological variation observed in the included papers was huge, making it difficult to draw conclusions from the body of the literature. For example, no two reported instances of reduced SDV were exactly alike. Similarly, the data processing and quality control applied to data prior to SDV were often not completely described. Significant differences were observed in available descriptions of upstream data processing, such as performing SDV against the medical record versus against structured site source worksheets. The contributions of SDV *versus* SDR (defined in Table 1) often could not be distinguished from one another. SDV includes reading the source record to ensure that there were no omissions in the study data which includes some amount of SDR. In fact, multiple included articles remarked that SDV was conducted by reading the full source. For this reason, findings attributed to SDV may have also been found or may be equally findable through SDR. Additionally, the variability in the terminology used in the literature could have easily led to inadvertent misclassification of studies with respect to the SDV process measured, the measures used, and the methods by which they were obtained.

## Conclusions

The review exposes significant heterogeneity in the SDV processes measured, the measures used, and the methods by which they were obtained. Though multiple studies quantified one or more aspects of SDV quality, we did not find an article that reported an assessment of SDV quality covering the full set of domains and levels identified in the included articles. Accuracy is not among the reported measures of SDV quality. Either the heterogeneity or the absence of accuracy measures alone is sufficient to preclude reporting, much less comparing, measurements of SDV quality found in the literature.

Due to the likely context sensitivity of SDV, QbD should be applied to ensure that new SDV approaches will not adversely impact human safety and research results. Additional research is needed to develop methods of designing study processes capable of delivering the necessary outgoing quality. Estimates of process and inspection accuracy are needed to support prospective process design.

The variability and error associated with SDV as a manual process suggest opportunities for improvement through advances such as automation and decision support.

## Supporting information

Hamidi et al. supplementary material 1 Hamidi et al. supplementary material

Hamidi et al. supplementary material 2 Hamidi et al. supplementary material

Hamidi et al. supplementary material 3 Hamidi et al. supplementary material

Hamidi et al. supplementary material 4 Hamidi et al. supplementary material

## Supplementary material

The supplementary material for this article can be found at https://doi.org/10.1017/cts.2024.551.
