# Supplementary material for: Source Data Verification (SDV) quality in clinical research: A scoping review
Source: J Clin Transl Sci. 2024 May 21;8(1):e101. doi: 10.1017/cts.2024.551 (PMC11639101; doi:10.1017/cts.2024.551)
Supplement: Hamidi et al. supplementary material 3 — Hamidi et al. supplementary material [file S205986612400551Xsup003.docx]

Appendix 3 Research Design and Context Detail for Included Quantitative Articles

| **Research Design** | **Error or Discrepancy Identification** | **Number of Studies**  **and Sites** | **Therapeutic Area** | **Industry Sponsor** | **Sample Size** | **Author’s Conclusion** |
| --- | --- | --- | --- | --- | --- | --- |
| **Experimental Designs** |  |  |  |  |  |  |
| Mealer *et al*. (2013) Prospective   |  | 2 Clinical Trials  5 sites | Acute respiratory distress; childhood liver disease | No | 32 Pts.  5954 data values  UOA: data value | Measured the difference in SDV findings between remote and on-site SDV of key variables. Concluded that SDV adds value. |
| Brosteanu *et al*. (2017) Prospective   |  | 11 Clinical Trials (low or intermediate risk)  213 sites  Multinational | - | No | 1618 Pts.  UOA: patient | Measured the difference in confirmed monitoring findings between 100% on-site SDV and risk-based (reduced) SDV. Concluded that, “compared with risk-adapted monitoring, the potential benefit of extensive on-site monitoring is small relative to overall findings.” |
| Fougerou-Leurent et al. (2019) Prospective   |  | 6 Clinical Trials  1 site  1 country | - | No | 126 Pts.  137,008 data values  UOA: data value | Measured data errors missed by on-site, targeted (reduced) SDV. Compared to the gold standard of 100% SDV, errors missed by on-site, targeted (reduced) SDV would not have impacted the outcome of included studies. |
| Engen *et al*. (2020) Prospective   |  | 1 Clinical Trial  196 sites  34 countries | Antiretroviral treatment in HIV | No | 4371 Pts.  UOA: patient | Measured the difference in monitoring findings between on-site monitoring and central + local monitoring. Concluded that, “the added value of on-site monitoring in START was minimal considering the cost.” |

| **Quasi-experimental Designs** |  |  |  |  |  |  |
| --- | --- | --- | --- | --- | --- | --- |
| Andersen *et al*. (2014). Retrospective   |  | 3 Clinical Trials | osteoarthritis  osteoporosis | Yes | 2566 Pts.  3,252,743 data values  UOA: data value | Measured the difference between the data discrepancies missed by 100% on-site SDV and no SDV. Concluded that, “the use of complete versus partial SDV offers a marginal absolute error rate reduction.” |
| Kondo et al. (2021) Prospective   |  | 1 Clinical Trial  11 sites  1 country (Japan) | Type 2 diabetes mellitus | Yes | 67 Pts.  UOA: data value | Measured the difference in data corrections found by 100% on-site SDV versus partial on-site SDV. Found that 100% on-site SDV following partial SDV identified no high-risk events. Concluded that, data corrections were comparable between partial and full SDV sites. |
| Andersen et al. (2022) ^ Retrospective   |  | 4 Clinical Trials  Same 3 sites in all four studies  1 country (Denmark) | osteoarthritis  osteoporosis | Yes | 2591 Pts.  UOA: data value | Measured differences in data discrepancies missed between RBM (targeted, reduced) SDV and the traditional approach. Concluded that the results, “suggest that RBM may improve data quality regarding data points of major importance… but observed variability in error rates using classic monitoring warrants caution in the interpretation”. |
|  |  |  |  |  |  |  |
| **Pre-experimental Designs** |  |  |  |  |  |  |
| Maruszewski *et al*. (2005). Prospective   |  | 1 Registry  5 sites  Multinational (European) | Congenital Heart Surgery | No | 1,703 Pts.  1.895 procedures  UOA: data value | Measured data and process problems found by on-sire SDV. Found no statistical differences between SDV’d and non-SDV’d data. Seven deaths were missed in non-SDV’d data. |
| Tudur Smith *et al*. (2012) Prospective   |  | 1 Clinical Trial  75 sites  1 country (United Kingdom) | Advanced cancer | No | 533 Pts.  UOA: data value | Measured discrepancies found and missed by SDV. Found that errors detected by SDV had little impact on the analysis, and that central monitoring was more effective for the overall survival outcome. Concluded that SDV was not error free. |
| TransCelerate (2013) Retrospective   |  | 9 Clinical Trials  6 sponsors | 8 Therapeutic areas | Yes | -  UOA: data value | Quantified SDV-identified discrepancies. Concluded that, “SDV has a negligible impact on data quality.” |
| Mitchel et al. (2014). Retrospective   |  | 1 Clinical Trial  18 sites  2 U.S. and Canada | - | Yes | 180 Pts.  5,581 source records  UOA: data value | Quantified SDV-identified data errors. Concluded that, “traditional SDV adds little value to support data quality and subject safety.” |
| Sheetz *et al*. (2014). Retrospective   |  | 1168 Clinical Trials  53 sponsors | Multiple therapeutic areas | Yes | -  UOA: data value | Quantified SDV-identified data errors and missed adverse events. Concluded that, “SDV has limited value as a primary quality management process” and that, on-site SDV or SDR “may be of value in identifying unreported events at some sites”. |
| Agrafiotis *et al*. (2018). Retrospective     |  | 12 Clinical Trials  - sites  - countries | - | Yes | -  UOA: audit findings | Quantified site quality control visit and audit findings tor trials employing risk-based monitoring (reduced SDV) or traditional monitoring (extensive SDV) and concluded that RBM with reduced SDV resulted in fewer critical and major findings from quality control and site audits. |
| Giganti *et al*. (2019). Retrospective   |  | 1 Registry  9 sites  Multinational (Caribbean, Central and South America) | HIV | No | 250 Pts.  14,489 data values  UOA: data value | Quantified data errors found by SDV, Concluded that, improved data quality following SDV may impact inferences. |
| Embleton-Thirsk *et al*. (2019). Retrospective   |  | 1 Clinical Trial  63 sites  multinational | Recurrent ovarian cancer | Yes | 390 Pts  66,947 data values  UOA: data value | Quantified SDV-prompted queries and SDV-identified toxicity grade errors. Concluded that, SDV, data audit and blinded central reading made immaterial changes to trial outcome measures. |
| Kim *et al*. (2021). Retrospective   |  | 3 Clinical Trials  1 site | Metastatic Colorectal Cancer | No | 74 Pts.  UOA: Monitoring findings | Quantified monitoring findings missed by central monitoring. Concluded that, “confirmatory SDV revealed more findings than central monitoring…” |
| Yamada *et al*. (2021). Retrospective   |  | 1 Clinical Trial  1 site  (Japan) | Chronic Kidney Disease | No | 11 Pts.  5,617 data values  UOA: data value | Quantified data error missed by remote SDV. Concluded that, on-site, 100% SDV added little value over remote, RBM. |

^The Andersen et al. (2022) study was published after the literature search period. We display it in the appendix for comparison only.

X: in a cell in the Error/Discrepancy Identification column signifies not measured or not reported.

M: in the Error/Discrepancy Identification column signifies that data values or events tallied in the indicated cells matched between the two compared data sources

NM: in the Error/Discrepancy Identification column signifies that data values or events tallied in the indicated cells did not match between the two compared data sources.

F: in the Error/Discrepancy Identification column signifies that data values or events tallied in the indicated cells were found in the compared data sources.

NF: in the Error/Discrepancy Identification column signifies that data values or events tallied in the indicated cells were not found in the compared data sources

C: in cells in a 2 x 2 table in the Error/Discrepancy Identification column signifies that the marked cells were collapsed (counted together) and reported as one number with individual contributing cell counts not reported.

NR in cells in a 2 x 2 table in the Error/Discrepancy Identification column signifies that the marked cells were stated as or implied to have been measured but were not reported.

* The cell was measured only for the parameter “death” through a third independent data source.
