# Supplementary material for: Source Data Verification (SDV) quality in clinical research: A scoping review
Source: J Clin Transl Sci. 2024 May 21;8(1):e101. doi: 10.1017/cts.2024.551 (PMC11639101; doi:10.1017/cts.2024.551)
Supplement: Hamidi et al. supplementary material 4 — Hamidi et al. supplementary material [file S205986612400551Xsup004.docx]

Appendix 4: Surveys

|  | Respondents | Focus | Major Findings Relevant to SDV Quality |
| --- | --- | --- | --- |
| Jochems *et al*. 1993 | Responses from 50 major pharmaceutical companies in Belgium, The Netherlands and the UK | “Attitudes of investigators and companies towards undertaking direct SDV”, “how much data companies verify in their clinical trials” and SDV methods | Most respondents had SOPs covering SDV. a smaller but still majority detailed SDV methods with the remainder leaving how SDV was to be carried out to the monitor’s judgement. SDV was performed at each site by roughly half of responding companies suggesting early risk-based approaches. |
| Hamrell *et al*. 2016 | The survey was publicized to Association of Clinical Research Professionals (ACRP) members. There were 199 responses, 88.9% of which were from the US. One fourth of the respondents reported working for study Sponsors with the remainder reporting working for sites. | “Utilization and considerations related to remote monitoring activities and their impacts on clinical data quality.” | When asked, “Do you believe that remote monitoring can have an impact on the quality of data collected for a clinical trial?”, 11.1% responded that they anticipated a positive impact, 26.6% responded that they didn’t anticipate any impact, and 62.3% responded that they anticipated a negative impact. |
| Hurley *et al*. 2017 | 132 academic clinical researchers working in Ireland were surveyed with a response rate of 49%. A sample of respondents were interviewed following the survey. | “To identify attitudes and perceived barriers and facilitators to the implementation of RBM in academic-led clinical trials.” | “Many [respondents] felt that sufficient proof did not exist to confirm that RBM was at least as effective and efficient as the 100% on-site monitoring process that they currently used.” “Many [respondents] felt that the merits of centralized monitoring had yet to be proven and so were not comfortable conducting RBM in future trials if it meant fewer on-site visits.” |
| Houston *et al*. 2018 | Identified contact persons from 142 clinical trials listed on the National Health and Medical Research Council (NHMRC) clinical trials website were invited to participate. The response rate was 14%. | To characterize current data quality tools and procedures used within Australian clinical trial sites. | The survey concluded that clinical trial sites used ad hoc methods to ensure data quality and that standardized data quality monitoring procedures were needed. |
| Kunzi *et al*. 2017 | Clinical Research Associates in Europe. | To determine how knowledge and practical experience with RBM have changed from 2014 (180 respondents) to 2016 (231 respondents). | The surveys reported “persisting skepticism regarding data quality in RBM studies” with 73% of RBM-experienced CRAs answering the question reporting that they did not believe RBM improves data quality in 2014 and slightly higher, 81% indicating such in 2016. Similarly, in 2014, 59% indicated a concern that SAEs might be overlooked with reduced SDV, and 64% indicated a similar in 2016. In both years 60% indicated a concern that protocol deviations might be overlooked with reduced SDV. |
| Funning *et al*. 2009 | The survey was distributed to 47 of the 60 member companies of the Swedish Association of the Pharmaceutical Industry (LIF) with a response rate of 62% covering 97% of the phase III clinical trials performed in Sweden in 2005. | To estimate the portion of phase III trial costs attributable to GCP-related activities. | “A solid majority (72%, n=28) did not agree that the present quality system (as applied by industry) for clinical trials is designed to guarantee a reliable scientific outcome. |
| Morrison *et al*. 2021 | The survey was distributed to 216 organizations involved in conducting clinical research with a 37% response rate. | To describe current methods of monitoring clinical trials and to explore rationale for the use of those methods. | On-site monitoring is routinely performed by industry and CROs even though it is widely accepted among Clinical Trial Transformation Initiative (CTTI) members that current on-site clinical monitoring practices do not always lead to increased quality in clinical trials and that “more research is need to assess the potential impact of the variations in the monitoring practices observed”. |
| Ward (2013) | The survey was distributed to 313 critical care investigators and research coordinators reported to be working with the Canadian Critical Care Trials Group with a response rate of 46.3%. | To describe attitudes and beliefs regarding source data verification within RCTs of the Canadian Critical Care Trials Group. | 98.5% of respondents indicated that SDV is an important part of QA, and 99.2% of respondents indicated that SDV helps ensure data reliability and validity while only 43.6% agreed that SDV was a cost-effective way of doing so. High percentages of respondents, ranging from 82.9% to 91.4%, indicted that evidence-based guidelines were needed for the amount and frequency of SDV and that more research was need on the effect of SDV on data validity, cost effectiveness of SDV, and the effect of SDV on study outcomes. |
